# Effect of integrated infectious disease training and on-site support on the management of childhood illnesses in Uganda: a cluster randomized trial

**DOI:** 10.1186/s12887-015-0410-z

**Published:** 2015-08-28

**Authors:** Peace Imani, Brian Jakech, Ibrahim Kirunda, Martin K. Mbonye, Sarah Naikoba, Marcia R. Weaver

**Affiliations:** Accordia Global Health Foundation, Washington, DC USA; International Training and Education Center for Health, Department of Global Health, University of Washington, Seattle, USA; Department of Pediatrics-Renal Section, Baylor College of Medicine, Houston, USA; Infectious Disease Institute, Department of Medicine, Makerere University, Kampala, Uganda; Elizabeth Glaser Pediatric AIDS Foundation, Kampala, Uganda; Departments of Epidemiology and Social Medicine, University of Antwerp, Antwerp, Belgium

**Keywords:** Mid-level practitioners, Clinical practice, Quality of health care, Education, Medical, Continuing, Mentors, Medicine, Infectious diseases, Training, Pediatrics, Primary health care, Africa, South of the Sahara, Uganda

## Abstract

**Background:**

The Integrated Infectious Disease Capacity-Building Evaluation (IDCAP) was designed to test the effects of two interventions, Integrated Management of Infectious Disease (IMID) training and on-site support (OSS), on clinical practice of mid-level practitioners. This article reports the effects of these interventions on clinical practice in management of common childhood illnesses.

**Methods:**

Two trainees from each of 36 health facilities participated in the IMID training. IMID was a three-week core course, two one-week boost courses, and distance learning over nine months. Eighteen of the 36 health facilities were then randomly assigned to arm A, and participated in OSS, while the other 18 health facilities assigned to arm B did not. Clinical faculty assessed trainee practice on clinical practice of six sets of tasks: patient history, physical examination, laboratory tests, diagnosis, treatment, and patient/caregiver education. The effects of IMID were measured by the post/pre adjusted relative risk (aRR) of appropriate practice in arm B. The incremental effects of OSS were measured by the adjusted ratio of relative risks (aRRR) in arm A compared to arm B. All hypotheses were tested at a 5 % level of significance.

**Results:**

Patient samples were comparable across arms at baseline and endline. The majority of children were aged under five years; 84 % at baseline and 97 % at endline. The effects of IMID on patient history (aRR = 1.12; 95 % CI = 1.04-1.21) and physical examination (aRR = 1.40; 95 % CI = 1.16-1.68) tasks were statistically significant. OSS was associated with incremental improvement in patient history (aRRR = 1.18; 95 % CI = 1.06-1.31), and physical examination (aRRR = 1.27; 95 % CI = 1.02-1.59) tasks. Improvements in laboratory testing, diagnosis, treatment, and patient/caregiver education were not statistically significant.

**Conclusion:**

IMID training was associated with improved patient history taking and physical examination, and OSS further improved these clinical practices. On-site training and continuous quality improvement activities support transfer of learning to practice among mid-level practitioners.

**Electronic supplementary material:**

The online version of this article (doi:10.1186/s12887-015-0410-z) contains supplementary material, which is available to authorized users.

## Introduction

Globally, 6.6 million children aged under five years died in 2012 [1]. Their leading causes of death in sub Saharan Africa included pneumonia, diarrhea, malaria and health problems during the first month of life [2]. Over two-thirds of these early childhood deaths could be prevented or treated with access to simple and affordable interventions [2]. Although the mortality rate among children aged under five years in Uganda declined from 178 per 1,000 live births in 1990 to 90 per 1,000 live births in 2011, it remains high compared to the average rate of 51 per 1,000 live births globally [3, 4]. Cross-country comparisons show that health outcomes improved and coverage of cost-effective interventions increased with more health workers. Mortality among infants and children under five years of age were inversely related to the total number of doctors, nurses, and midwives per 10,000 population, and vaccination coverage was positively related to it [5, 6].

The World Health Organization (WHO) recommends task shifting as one method of strengthening and expanding the health workforce to rapidly increase access to health services [7, 8]. Task shifting is a process of delegation of tasks, where appropriate, to less specialized health workers and is a strategy to address health worker shortage and improve health care coverage [7, 9]. In countries with an inadequate healthcare work force, some clinical responsibilities have been transferred from doctors to mid-level healthcare providers such as clinical officers and registered nurses [8, 10, 11]. Policy discussions about task shifting or sharing have moved from whether or not clinical officers and registered nurses can effectively perform clinical tasks traditionally reserved for doctors to what are the most effective methods for improving their capacity to perform those tasks [12, 13].

Evidence from the Integrated Management of Childhood Illness (IMCI) Multi-Country Evaluation showed that training improved the quality of care, but there was room for further improvement [14–18]. Among health workers with IMCI training, the quality of care was better with at least one supervision visit every six months in Uganda [14), and with study supports including supervision in Benin (19]. Horwood et al. (2009) recommended further research on the role of supervision to maintain IMCI skills and on different models of supervision [20]. The Joint Uganda Malaria Training Program (JUMP) combined classroom sessions, practice and supervision visits, and was effective at improving case management of fever among children [21]. Several of these facilities achieved high levels of performance after four years of ongoing site visits with data surveillance and feedback [22].

The objectives of the Integrated Infectious Disease Capacity Building Evaluation (IDCAP) were to design two integrated training interventions for mid-level practitioners (MLP), evaluate their effectiveness, and estimate their cost-effectiveness at 36 facilities in Uganda. The two interventions were an Integrated Management of Infectious Diseases (IMID) training program, and on-site support (OSS). The effects of the interventions on the clinical competence and clinical practice of individual MLP, 23 facility performance indicators, and mortality among children under five years of age were tested. Clinical competence measured by vignettes, which are sometimes referred to as case scenarios, increased significantly after the 3-week core IMID course and persisted for 24 weeks; no incremental effect of OSS was observed [23]. Similarly IMID was associated with statistically significant improvements in two facility performance indicators, and the combination of IMID and OSS were associated with statistically significant improvements in five [24, 25]. Despite large incremental effects of OSS on 10 indicators, none were statistically significant. The results for clinical practice in management of common childhood illnesses are reported below.

## Methods

### Trial design

The evaluation of clinical practice was conducted between January 2010 and February 2011, and had a mixed design with pre/post and cluster-randomized trial components.. Thirty-six eligible health facilities were selected from all major regions of Uganda [26] and randomized as clusters, because many of the facility performance indicators depended on a team of clinicians, laboratory professionals and other staff rather than individuals. Each facility selected two MLP, who were clinical officers, registered nurses, or registered midwives to participate in the IMID training program. The clinical officers had a secondary school education, three years of pre-service training, and two years of internship. The registered nurses and registered midwives had a secondary school education, and three years of pre-service training. Eighteen health facilities were then randomly assigned to receive OSS (arm A) in 2010 while the other 18 facilities received it in 2011 and served as a control for OSS during the trial (arm B). The full protocol is available as a supplementary file for Mbonye et al. [24] and Weaver et al. [25]. The CONSORT Checklist for the trial is in Additional file 1.

### Facilities

The health facilities comprised of 31 Health Centers IV and five general hospitals. These facilities served a subdistrict population of 1.39 million or 4.5 % of the 30.66 million projected population in 2009 [24, 25, 27]. Two key inclusion criteria were: 1) facilities were accredited to provide anti-retroviral therapy (ART) to patients with HIV, and 2) facilities with a functional laboratory defined as performing six tests: malaria blood smear, human immunodeficiency virus (HIV) rapid test, tuberculosis (TB) sputum smear, urinalysis, stool analysis, and haemoglobin estimation. For more details on inclusion criteria for health facilities see Miceli et al. [28] and Naikoba et al. [26].

### Trainees

The MLP trainees were clinical officers (CO) and registered nurses (RN) who managed patients in the outpatient clinics, devoted at least 80 % of their time to patient care, and were available to participate in the evaluation for 21 months [26, 28]. The district agreed not to transfer the trainee, and the trainee did not foresee a need to change his/her assignment. If a facility did not have two COs who met these criteria, an RN was selected and if an RN was not available, one trainee could be a registered midwife. Two desirable criteria for trainees were: 1) leadership role such as in-charge of ward or clinic, or focal person for malaria, TB, HIV, prevention of mother-child transmission of HIV (PMTCT), and 2) previous training and experience in counseling or IMCI or Integrated Management of Adult Illness (IMAI). For the clinical assessments, trainees were excluded from the analysis if they were observed managing only patients 14 years of more of age.

### Patient selection

Patients were a convenience sample who reported to the clinic on the day of the assessment was conducted by the clinical faculty. Patients under five years of age were preferred, but older children were selected when there were no younger patients at the facility on the day of the assessment.

### Interventions

IMID training program was a three-week core course, followed over a nine-month period by distance learning and two one-week boost courses at 12 and 24 weeks after the initial training. The aim of IMID was to improve diagnosis and management of malaria, TB and HIV/AIDS and related infectious diseases in children, pregnant women, and other adults. The course content was guided by learning need assessments, such as Lutalo et al. [29]. It built on content from WHO’s IMAI [30] and IMCI [31] courses; IDI’s Comprehensive HIV Care including ART [32] and Joint Uganda Malaria Training Program (JUMP) [21] courses, and updated national and WHO guidelines on treatment of malaria, TB and HIV/AIDS. It also included sessions on continuous quality improvement [33], with adaptations for low and middle income countries. Detailed description of the development and content of the IMID course is in Miceli et al. [28]. Between courses, trainees continued the learning process by reviewing and documenting 10 case write-ups from a menu linked to material covered in the core course. Technical experts based at IDI provided distance support on request for all trainees. Trainees in arm A attended the IMID core course in March and April 2010 while those in arm B attended in May and June 2010.

The OSS intervention combined educational outreach and continuous quality improvement (CQI). A mobile faculty team visited each facility in arm A for two days, once a month for a period of nine months. Each mobile faculty team was comprised of a medical officer with CQI expertise, a clinical officer, a nursing officer, and a laboratory technologist. OSS sought to improve individual practice through clinical mentoring. At each of the facilities in arm A, eight clinicians spending 80 % of their time on patient care were selected for one-to-one mentoring by two clinical faculty (medical officer and clinical officer) while the laboratory staff were mentored by the laboratory technologist. OSS also sought to build and foster team work, improve facility performance, and support the use of data to monitor facility performance through multidisciplinary team (MDT) training, cadre-specific clinical breakout sessions, and CQI activities. All clinical staff were invited to participate in the MDT training sessions and their respective cadre specific break-out sessions. The break-out session for clinicians may have also contributed to improving clinical practice. IMID trainees in arm A were required to attend seven of nine OSS sessions to receive a certificate. During each OSS visit, the MDT and cadre-specific sessions were focused on a selected topic from a predefined list of priority areas. A new topic was addressed each visit, and follow-up support on topics covered in the previous visits was also provided. OSS is described in more detail in Miceli et al. [28] Naikoba et al. [26] and Mbonye et al. [24].

The mobile faculty team acted as role models of how an MDT could function. The team was centrally based to avoid contamination of non-intervention facilities, which could have occurred if the Ministry of Health (MoH) mentors from the district offices who had other responsibilities at facilities in both arms were used. However, for sustainability purposes and to address health system issues at the district level, the nursing officer was selected from the district in which the facility was located. There were five mobile faculty teams, each responsible for 3–4 study facilities located in the same or neighboring geographical region.

The medical officers and clinical officers on the mobile faculty team also conducted the clinical assessments. They underwent extensive training prior to the baseline assessments and delivering the OSS sessions. They attended IDI’s three-week Comprehensive HIV Care including ART therapy course, a Trainer of Trainers course, and a customized two-week course on mentoring. All members of the mobile faculty team, including representatives of the districts, attended a pilot session of IMID.

### Pre-and post-intervention assessment

A “baseline” assessment was conducted between January and March 2010 prior to any of the interventions and is referred to as time 0. An “endline” clinical assessment was conducted from December 2010 to February 2011, beginning during the ninth OSS visit in arm A, and is referred to as time 1. At both time 0 and time 1, trainees were assessed on a single day on at least five patients at each facility in each time period.

### Outcomes

The clinical practice outcomes were six sets of tasks: 1) Appropriate patient history questions asked given the patient’s presenting symptoms; 2) Physical systems examined correctly during examination; 3) Appropriate laboratory tests; 4) Correct diagnosis; 5) Correct treatment (anti-malarials, antibiotics, or other drugs), and 6) Appropriate education regarding the illness and medication prescribed provided to the patient or caregiver.

A pretested standardized assessment tool was used to record trainees’ clinical practice and anonymous patient information on these six sets of tasks. The tool was based on previous IMCI and JUMP evaluation tools with two important innovations from the work of Brentlinger et al. [34–37]. The clinical faculty teams: 1) recorded information on patients as well as trainee practice so accurate patient information was available, and 2) interrupted the assessment after the trainee completed the patient history and physical examination. This was done to ask additional history questions and conduct additional physical examination to complete missing information or correct erroneous information. Then the assessment of the trainee continued. History questions included follow-up questions for patients who presented with fever, ear discharge, cough, and diarrhea to establish duration and extent of illness. Consequently the required number of history questions varied across patients according to their symptoms. To distinguish patient information obtained by the trainee from that obtained by the faculty on the same form, trainees’ clinical findings were recorded in blue or black ink and the faculty members’ were recorded in red ink.

### Construction of outcome variables

#### History

Trainees were expected to ask patients or caregivers questions and record correct responses for at least nine main questions about: danger signs, fever, ear discharge, cough, diarrhea, immunization status, HIV status of the child or mother if child’s status was not known, and other symptoms. Table 1 shows the main questions and respective follow up questions. The number of appropriate questions depended on the patient’s symptoms and ranged from eight to 19. The number of appropriate questions comprised the denominator while the numerator was the number of actual questions the trainee asked. In the analysis, we modelled the proportion of appropriate questions asked for any given patient.

**Table 1:** Patient history fields on which trainees were assessed

Main history questions		Follow up questions
1	Checked for danger sign	
2	Checked for fever	If fever is present
		2a. Asked about duration
		2b. Asked about prior antimalarial use
		2c. Asked about history of measles within last 3 months
		2d. Asked about history of ear pain
3	Checked for ear discharge	If ear discharge is present
		3a. Duration
4	Asked about HIV status	
5	Checked for cough	If cough is present
		5a. Asked about duration
		If cough duration >14 days
		5b. Asked for history of night sweats
		5c. Asked for history of weight loss
		5d. Asked for history of contact with patient with TB
6	Checked for diarrhea	If diarrhea is present
		6a. Asked about duration
		6b. Asked about presence of blood in stool
7	Checked for immunization (children < 5y)	
8	Checked for other problems	

#### Physical examination

For each patient assessed, trainees were expected to perform a physical examination consisting of eight systems: a general examination, check for danger signs, and examination of the respiratory system, abdomen, ears, skin, basic neurological assessment, and check for any other clinical signs. These eight systems comprised the denominator for the physical examination. Growth assessment was not included because of missing data, and other systems were only included when data were not missing. The numerator was the number of systems examined correctly by the trainee, where correct meant both conducting the examination and identifying the physical symptom or its absence. In the analysis, we modelled the proportion of physical systems examined correctly for any given patient.

#### Laboratory tests, diagnosis, treatment and patient/caregiver education

Laboratory tests were measured as a single task meaning that all appropriate tests were ordered, and diagnosis measured as a single task meaning that all diagnoses were correct. Treatment was measured as the proportion of three types of treatment prescribed correctly: malaria, antibiotic, and other treatment. Similarly, patient/caregiver education was measured as the proportion of three types of information provided correctly: diagnosis, treatment, and instructions for completing treatment. Incorrect treatment included both errors of omission (e.g. a malaria test was not ordered for a patient with fever) and errors of commission (e.g. an anti-malarial was prescribed for a patient with a negative malaria test result).

#### Changes after trial commencement

The patient sample and the assessment tool changed. When some facilities did not have five patients under five years of age on assessment days, a few trainees were assessed on children over five years.

The assessment tool was also revised prior to conducting the endline assessment to address problems with the patient history and physical examination sections identified at baseline. The patient history questions about HIV status and immunization were revised. At baseline, it was unclear whether “yes”, meant that the child or mother was positive or that the HIV status was known. Immunization status of the child was similarly unclear as to whether “yes” meant that the immunization status of the child was up-to-date or that the child’s immunization card was presented. For both tasks, a “yes” at baseline was interpreted to mean that the trainee spoke with the caregiver about the topic. At endline there were three distinct tasks for asking about mother’s HIV status, PMTCT, and child’s HIV status. A “yes” for the child’s status meant that the child was HIV-positive. For immunization status at endline, there were two distinct tasks for asking: 1) Whether or not the child’s immunization status was up to date, and 2) Whether or not the immunization status was verified with an immunization card. Physical examination questions about the mouth were added to the form. The endline version of the assessment form is available for researchers as Additional file 2. Data on HIV status, immunization and examination of the mouth were only included in the sensitivity analysis performed with the endline sample. (See Sensitivity analysis section below).

#### Sample size

The sample size calculations for IDCAP were based on testing the effect of OSS on facility performance where the unit of analysis was the health facility. The calculations were reported in Naikoba et al. [26].

Feasibility rather than sample size calculations guided the number of IMID trainees per facility and number of patients observed per trainee. The initial proposal was to train all the MLP at each facility, based on evidence of the effect of other IDI training programs on clinical practice [21, 32]. Funding was only available however, to train two MLP per facility. Similarly, clinical faculty teams could only spend one day per facility on the clinical assessments of pediatric care, and early experience at baseline showed that each faculty member could observe five patients per trainee per day. Given 36 trainees per arm and five observations per trainee, we planned to have a sample of 180 observations per arm each time period. In a simple comparison of proportions at time 1, a sample size of 180 would detect an increase from 60 % to 75 % of tasks performed correctly with a power of 0.84 and an increase from 70 % to 85 % of tasks performed correctly with a power of 0.91.

#### Randomization—sequence generation

Health facilities were assigned to arms by stratified random allocation [23, 26]. Facilities were stratified by two characteristics: 1) prior experience with the Health Care Improvement program, a national CQI program for HIV prevention and treatment vs. CQI naïve, and 2) current or prior participation in the Baylor International Pediatric AIDS Initiative (BIPAI) on-site intervention, vs. no BIPAI presence [38]. Facilities were then randomly assigned to arm A or B (1:1 balance) within those strata.

Randomization of health facilities to arm was implemented using random number generation in Stata 10.1. We retained a log of the Stata run stream that led to the randomization and set the “seed” from which the randomization started so that it could be replicated if the need arose.

#### Randomization—allocation concealment

Randomization occurred on February 23, 2010 after the majority of trainees completed baseline clinical assessments in January and February 2010. Within 2 weeks of the first session of the IMID course, arm A trainees were notified of their upcoming course dates and arm assignment. Allocation was not concealed during the interventions and endline assessment.

Clinical faculty member who conducted baseline assessment at a facility may have subsequently been assigned to provide OSS at that facility. To minimize observer bias in favor of mentees, endline assessments were conducted by a clinical faculty member who did not provide OSS at the facility [39]. However, observer bias to favor arm A trainees may have existed.

#### Randomization—implementation

Trainees were assigned to interventions based on the allocation of their facility to arm*.*

#### Blinding

This study was not blinded after randomization in February 2010.

#### Data management and statistical methods

Data were coded by a Ugandan medical doctor (PI), entered in Epiinfo3.2® (U.S. Centers for Disease Control and Prevention, Atlanta GA), cleaned and validated. Baseline data were entered once and proofread by PI. Endline data were double entered. All analyses were performed with Stata 11 (StataCorp, 2009 College Station, TX).

Trainee and patient characteristics at baseline were summarized. To test the effect of the intervention on each outcome, the pre/post (endline vs. baseline) difference in arm B measured the effect of IMID, and the difference in the pre/post difference between arm A and B measured the incremental effect of OSS. The patient was the unit of analysis. For each outcome, patient and time point, we observed a binomial count of the number of tasks performed correctly, where the total number of appropriate tasks varied across patients for patient history and physical examination. We used a generalized linear model, specifically a Poisson family and log link, with main effects for arm, time period and their interaction to estimate relative risks (RR) and ratio of relative risks (RRR). Although patients were observed with random effects for trainee nested within health facility, preliminary analyses suggested that the random effect for facility did not affect the results, and was not included in the primary model reported below. All regression analyses were clustered on the trainee with robust variance estimation to adjust for using the Poisson rather than the binomial family and for over-dispersion. The results for the interventions were presented as adjusted RR (aRR) for the effect of IMID, and adjusted RRR (aRRR) for the incremental effect of OSS, with 95 % confidence intervals (95 % CI). A 5 % level of significance was used with the caveat that there were multiple comparisons, which increased the chance of a Type I error, i.e. the probability of erroneously concluding that there was an effect of the IDCAP interventions.

To address any residual confounding after randomization, we adjusted for several covariates: trainee profession, the two strata (CQI experienced and presence of BIPAI at the facility), and learning during the assessment, which was measured using a categorical variable taking on values of one to five for the sequence of the particular patient being observed for each trainee. We also adjusted for case complexity with two variables: 1) two or more diagnoses, and 2) patient less than one year of age. We intended to include a fixed effect for observer, but it was not possible for the primary model, because of the imbalance of clinical faculty across facilities and time periods (see Sensitivity analysis section below). Two alternative variables to adjust for observer were used: 1) the profession of faculty member (medical officer or clinical officer), and 2) whether or not the faculty member attended the assessment training session for the time period during which the assessment was conducted.

Several sensitivity analyses were performed with different subsamples and models. Analyses were repeated with the subsample of patients with clinical faculty members balanced across facilities and time periods including a fixed effect for observer instead of the alternate variables described above. This subsample only included observations of trainees by the clinical faculty members who conducted assessments at baseline (time 0) and endline (time 1), and with trainees in both arm A and arm B during each time period. This is referred to as the balanced sample in the results below while analysis of all clinical assessments is referred to as the full sample.

Given the comparable practice across arms A and B at baseline, analyses were repeated with endline data only to take advantage of improvements in the assessment form with experience. Finally, regression diagnostics were performed to identify outliers and influential observations and estimates were obtained excluding these observations.

The primary model was estimated with complete cases for each set of tasks, meaning that information was reported on each task within a set. For patient history, two tasks were not included in the full and balanced sample comparisons, because data were missing for 30 or more patients: checked for danger signs, and measles within last three months. (See Additional file 3.) For physical examination, examination of growth was not included in the full and balanced sample comparisons for the same reason. (See Additional file 4.) In addition, “other” system was not included in the numerator or the denominator when data about it were missing. Sensitivity analyses were performed with two alternative assumptions about missing values for the remaining sets of tasks: 1) all missing values were interpreted as the task not performed by the trainee, i.e. missing values were equal to zero, and 2) all missing values were interpreted as performed by the trainee, i.e. missing values were equal to one.

#### Human subjects’ approval

IDCAP was reviewed and approved by the School of Medicine Research and Ethics Committee of Makerere University (Reference number 2009–175) and the Uganda National Committee on Science and Technology (Reference number HS-722). The University of Washington Human Subjects Division determined that the study did not meet the regulatory definition of research under 45 CFR 46.102(d).

IMID trainees were asked to provide written informed consent for secondary analysis of their training program data for the evaluation. Patients and their caregivers were introduced to the assessments in the waiting area on the day of the assessment and asked to provide verbal informed assent/consent at the beginning of the consultation. The patient data were anonymous, and the verbal consent process preserved their anonymity.

#### Recruitment of facilities and trainees

The facilities were recruited between March and September 2009. The registration and consent process for the IMID training trainees took place between December 2009 and March 2010.

## Results

### Facility and trainee flow

Thirty-six health facilities (five hospitals and 31 health centers) enrolled in this evaluation and participated throughout. Four of the five hospitals were randomized to arm B. A total of 72 MLP were selected to participate in IMID. The MLP comprised of 48 clinical officers, 20 registered nurses and 4 registered midwives. All four registered midwives were based at facilities randomized to arm B. Seventy-two percent and 61 % of MLP were Clinical Officers in arm A and arm B respectively. As noted above, we controlled for trainee cadre in the analysis to adjust for potential residual confounding.

**Fig. 1:**
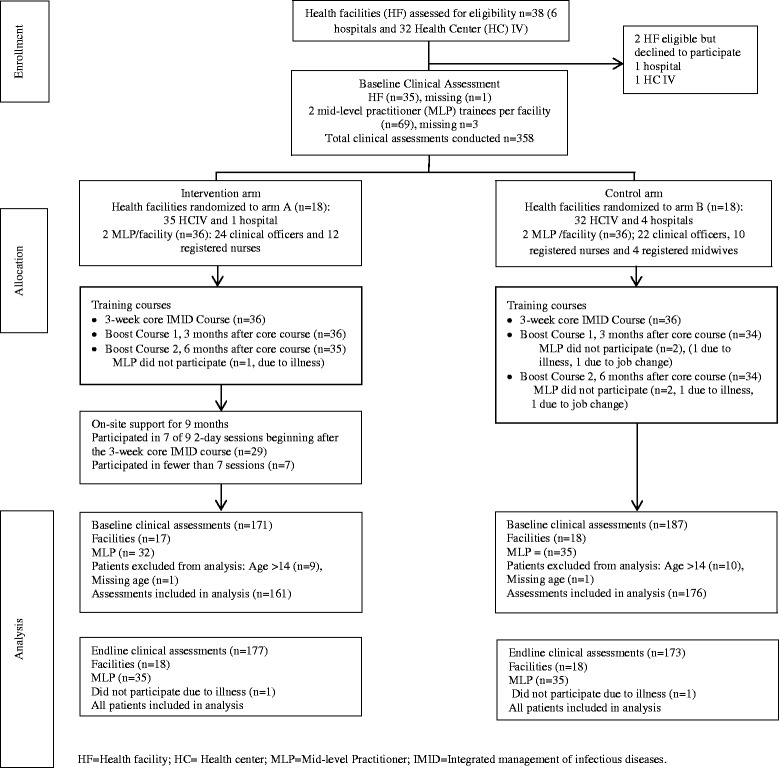
Flow diagram of facilities and trainees

A baseline clinical assessment was conducted at 35 facilities with 69 MLP. Assessments forms for one of the facilities were unavailable, and one MLP was recruited after the baseline clinical assessments were completed. One MLP who only assessed children aged more than 14 years was excluded from the analysis, and one was excluded for a reason that was not documented. Randomization was followed by the three-week IMID core course for all 72 MLP and follow up boost courses (Fig. 1). Four MLP, one from arm A and three from arm B were not available to participate in at least one booster course. An endline clinical assessment was conducted at all 36 facilities with 70 MLP. A total of 687 (337 baseline and 350 endline) patients were included in the analysis. There were some incomplete data for the patient history, physical examination, and laboratory tests as shown by the sample sizes in Table 3. Details of the missing data are in Additional files 3 and 4.

### Baseline data

Arm A and B facilities were distributed relatively evenly across geographic regions, with the exception that arm A included disproportionately more facilities in the southwest region and arm B included disproportionately more facilities in the central region. Seventeen of the 36 facilities had previously participated in a national CQI program for HIV care and 10 were previous or current participants in a BIPAI on-site intervention; eight and five, respectively in arm A, nine and five, respectively in arm B.

The characteristics of the patient sample are described in Table 2. The most common presenting complaints were fever and cough, while the most frequent diagnoses were uncomplicated malaria and cough (no pneumonia). Three percent and 2 % of the patients were classified as emergencies at baseline and endline, respectively, even though clinical faculty were instructed not to use them in the assessment of trainees.

**Table 2:** Descriptive characteristics of patients by time and arm

	Baseline			Endline		
	Arm A	Arm B	Total	Arm A	Arm B	Total
	*N* = 161	*N* = 176	*N* = 337	*N* = 177	*N* = 173	*N* = 350
Characteristic	N (%)	N (%)	N (%)	N (%)	N (%)	N (%)
Age						
<5	130 (81)	153 (87)	283(84)	174 (98)	166 (96)	340 (97)
5-14	30 (19)	22 (13)	53 (16)	3 (2)	7 (4)	10 (3)
Triage Status						
Emergency	3 (2)	6 (4)	9 (3)	1 (1)	4 (3)	3 (2)
Priority^b^				22 (14)	3 (2)	25 (8)
Not emergency	150 (98)	157 (96)	307 (97)	132 (85)	152 (96)	284 (90)
Febrile or AT > 37.5 °C	82 (55)	89 (54)	171 (54)	65 (37)	49 (28)	114 (33)
Type of visit						
New attendance	144 (94)	155 (95)	299 (94)	172 (99)	166 (97)	338 (98)
Re-attendance	10 (6)	9 (5)	19 (6)	2 (1)	6 (3)	8 (2)
Patient symptoms						
Fever	131 (82)	154 (88)	285 (85)	151 (86)	143 (84)	294 (85)
HIV exposed	3 (2)	1 (1)	4 (1)	3 (2)	2 (1)	5 (1)
HIV-status^a^	27 (20)	35 (22)	62 (21)	6 (4)	4 (3)	10 (3)
Cough	91 (57)	130 (74)	221 (66)	122 (69)	114 (67)	236 (68)
Diarrhea	48 (30)	52 (30)	100 (30)	44 (25)	48 (28)	92 (26)
Vomiting	29 (18)	32 (18)	61 (18)	24 (14)	36 (21)	60 (17)
Ear pain	11 (7)	6 (4)	17 (5)	11 (7)	6 (4)	17 (5)
Ear discharge	9 (6)	4 (2)	13 (4)	8 (5)	3 (2)	11 (3)
Diagnoses						
One or more danger signs	0 (0)	2 (1)	2 (1)	3 (2)	2 (1)	5 (1)
Anemia	10 (6)	19 (11)	29 (9)	20 (11)	22 (13)	42 (12)
Cough (no pneumonia)	58 (36)	65 (37)	123 (36)	72 (41)	66 (38)	138 (39)
Pneumonia	29 (18)	37 (21)	66 (20)	33 (19)	23 (13)	55 (16)
Diarrhea – acute	24 (15)	30 (17)	54 (16)	19 (11)	26 (15)	42 (13)
Ear infection	12 (7)	4 (2)	16 (5)	9 (5)	8 (5)	17 (5)
Malaria (uncomplicated)	74 (46)	83 (49)	160 (47)	61 (34)	62 (36)	123 (35)
Malaria (complicated)	21 (13)	33 (19)	54 (16)	15 (8)	19 (11)	34 (10)
Malnutrition (LWA)	10 (6)	25 (14)	35 (10)	18 (10)	9 (5)	27 (8)
Severe malnutrition	3 (2)	1 (1)	4 (1)	4 (2)	3 (2)	7 (2)
UTI	6 (4)	10 (6)	16 (5)	3 (2)	8 (5)	11 (3)
Number of diagnoses						
1	62 (39)	54 (31)	116 (35)	72 (41)	72 (41)	147 (42)
2	64 (40)	62 (35)	126 (38)	69 (39)	66 (38)	135 (39)
>2	33 (21)	60 (34)	93 (28)	36 (20)	32 (19)	68 (19)

^a^ At baseline, reflects children and mothers (of children less than 5 years) whose HIV status was known regardless of results while at endline, it reflects those who were HIV positive. For trainee assessment, of interest was whether the trainee made an effort to ask or not. AT = axillary temperature, LWA = low weight for age

^b^ Not included on the baseline assessment tool

### Outcomes and estimation

**Fig. 2:**
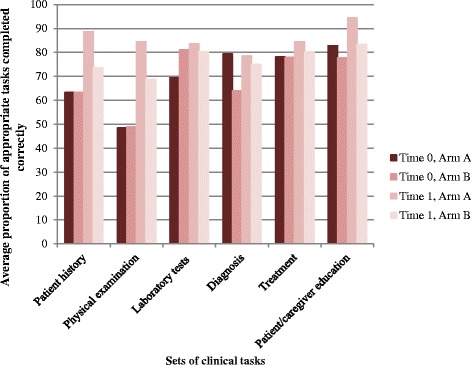
Unadjusted average proportion of appropriate tasks performed correctly by arm and time period

Figure 2 shows the unadjusted average proportion of appropriate tasks performed correctly comparing arms A and B at baseline (time 0) and at endline (time 1). Baseline clinical practice between arms A and B was comparable. Arm A had higher scores at endline than arm B although these average proportions were not adjusted for confounding variables.

Regression results for the full sample are shown in Table 3. There was no statistically significant difference in the practice of MLP in arm A compared to arm B at baseline (time 0), but the percentage of appropriate laboratory tests ordered was lower (aRR = 0.85 (95 % CI = 0.72–1.01)) and the percentage of correct diagnoses was higher (aRR 1.14 (95 % CI = 0.98-1.33)) in arm A than arm B. For the primary model, the multivariate analysis controls for differences across arms at baseline. Clinical practice of MLP improved after IMID in arm B. Adjusted for covariates, there was a 12 % increase in the proportion of appropriate history questions asked (aRR = 1.12 (95 % CI = 1.04–1.21)) and a 40 % increase in the proportion of physical systems examined correctly (aRR = 1.40 (95 % CI = 1.16–1.68)). The aRRs within arm B for laboratory tests, diagnosis, treatment, and patient/caregiver education were close to one and not statistically significant.

**Table 3:** Primary analysis: Adjusted relative risks (95 % confidence intervals) for performing tasks correctly for the full sample across arms and time periods

	Sets of clinical tasks					
Effects	Patient history	Physical examination	Laboratory tests	Diagnosis	Treatment	Patient/caregiver education
Sample Size	*N* = 573	*N* = 639	*N* = 621	*N* = 674	*N* = 683	*N* = 667
Arm A vs. Arm B at time 0	1.02 (0.93-1.11)	0.97 (0.80-1.18)	0.85 (0.72-1.01)	1.14 (0.98-1.33)	1.00 (0.92-1.09)	1.07 (0.95-1.19)
Time 1 vs. time 0 in Arm B (IMID)	1.12 (1.04-1.21)	1.40 (1.16-1.68)	0.95 (0.82-1.09)	1.05 (0.91-1.21)	1.01 (0.93-1.10)	1.03 (0.92-1.16)
Change Arm A vs. Arm B (IMID and OSS), RRR	1.18 (1.06-1.31)	1.27 (1.02-1.59)	1.21 (0.97-1.49)	0.92 (0.77-1.10)	1.07 (0.96-1.19)	1.07 (0.94-1.23)

Estimates were adjusted for: sequence of clinical assessment, cadre of trainee, complexity of patient determined by number of diagnoses and age of patient less than one year, whether the health facility received support from the Health Care Improvement project or not, whether the facility received the on-site intervention from Baylor International Pediatric AIDS Initiative or not, and the clinical faculty determined by cadre of faculty and whether s/he attended relevant assessment training session or not

RRR = Ratio of relative risks comparing change in practice at time 1 to practice at time 0 across arms

The incremental effect of OSS was measured by the ratio of the relative risks of outcomes at baseline to endline by trainees in arm A compared to the corresponding relative risks in arm B. As shown in the last row of Table 3, adjusted for covariates, OSS was associated with an additional 18 % (aRRR = 1.18 (95 % CI = 1.06–1.31)) improvement in patient history and 27 % (aRRR = 1.27 (95 % CI = 1.02-1.59)) improvement in physical examination. The percentage of patients with appropriate laboratory tests increased by 21 % (aRRR = 1.21 (95 % CI = 0.97–1.49)), but the effect was not statistically significant. Estimated effects for diagnosis, treatment, and patient/caregiver education were smaller and not statistically significant.

### Sensitivity analyses

Two clinical faculty members at baseline who were not able to participate in the endline assessment were replaced by two new members. Two additional clinical faculty present at baseline were only available to conduct half of their endline assessments and were replaced with two additional members for a total of four new clinical faculty members at endline. Sensitivity analyses were conducted using a balanced sample that consisted of assessments by the eight clinical faculty who were present both at baseline and at endline and who conducted roughly the same number of assessments in each arm at each time period.

The balanced sample comprised a total of 58 trainees (29 in arm A and 29 in arm B) and 440 clinical assessments (206 from arm A and 234 from arm B). There were 215 and 225 clinical assessments at baseline and at endline respectively. As shown in Table 4, baseline practice was comparable across arm A and arm B. Adjusted for covariates, IMID was not associated with improvements. OSS was associated with an additional 31 % (aRRR = 1.31 (95 % CI = 1.13–1.53)) improvement in patient history. The improvements in physical examination (aRRR = 1.27 (95 % CI = 0.99–1.63)) and laboratory tests (aRRR = 1.24 (95 % CI = 0.99–1.54)) were large, but not statistically significant.

**Table 4:** Sensitivity analysis: Adjusted relative risks (95 % confidence intervals) of performing task correctly for the balanced sample* across arms and time periods

	Sets of clinical tasks					
Effects	Patient history	Physical examination	Laboratory tests	Diagnosis	Treatment	Patient/caregiver education
Sample Size	*N* = 344	*N* = 404	*N* = 397	*N* = 435	*N* = 438	*N* = 425
Arm A vs. Arm B at time 0	0.96 (0.86-1.08)	1.05 (0.87-1.26)	0.87 (0.74-1.02)	1.06 (0.90-1.24)	1.05 (0.97-1.15)	1.04 (0.92-1.18)
Time 1 vs. time 0 in Arm B (IMID)	1.00 (0.92-1.10)	1.15 (0.93-1.43)	0.86 (0.74-1.01)	0.86 (0.73-1.00)	0.96 (0.87-1.06)	1.03 (0.91-1.17)
Change Arm A vs. Arm B (IMID and OSS), RRR	1.31 (1.13-1.53)	1.27 (0.99-1.63)	1.24 (0.99-1.54)	1.17 (0.96-1.44)	1.08 (0.96-1.21)	1.06 (0.91-1.24)

*Balanced consists of observations for whom the clinical faculty at baseline and endline were the same, with a fixed effect for the observer

Estimates were adjusted for: sequence of clinical assessment, cadre of trainee, complexity of patient determined by number of diagnoses and age of patient less than one year, whether the health facility received support from the Health Care Improvement project or not, whether the facility received on-site intervention from Baylor International Pediatric AIDS Initiative or not

RRR = Ratio of relative risks comparing change in practice at time 1 to practice at time 0 across arms

Given there were no statistically significant differences in clinical practice across arms at baseline, we conducted an analysis using only the endline sample, comparing arm A to arm B to take advantage of additional variables that were available only at endline. Using the full sample at endline, OSS was associated with improved clinical practice across all sets of tasks assessed, but improvement was not statistically significant for laboratory tests and diagnoses (See Table 5). In addition to improvement in patient history (aRR = 1.24 (95 % CI = 1.14–1.35)) and physical examination (aRR = 1.26 (95 % CI = 1.12–1.41)), there were effects of OSS on prescribing of appropriate treatment (aRR = 1.08 (95 % CI = 1.01–1.16)) and patient/caregiver education (aRR = 1.13 (95 % CI = 1.02–1.25)).

**Table 5:** Sensitivity analysis: Adjusted relative risks (95 % confidence intervals) of performing task correctly for the endline sample across arms

Sets of clinical tasks						
Effects	Patient history	Physical examination	Laboratory tests	Diagnosis	Treatment	Patient/caregiver education
Sample Size	*N* = 312	*N* = 350	*N* = 323	*N* = 345	*N* = 350	*N* = 346
Arm A vs Arm B at time 1	1.24 (1.14-1.35)	1.26 (1.12-1.41)	1.02 (0.91-1.15)	1.10 (0.95-1.28)	1.08 (1.01-1.16)	1.13 (1.02-1.25)

Two additional patient history tasks were asking for presence of danger signs and measles status. Two additional physical systems examined were mouth and growth assessment

Estimates were adjusted for: sequence of clinical assessment, cadre of trainee, complexity of patient determined by number of diagnoses and age of patient less than one year, whether the health facility received support from the Health Care Improvement project or not, whether the facility received on-site intervention from Baylor International Pediatric AIDS Initiative or not, and the clinical faculty determined by cadre of faculty and whether s/he attended relevant assessment training session or not

Sensitivity analyses were conducted with imputation of missing data on the clinical tasks. In estimates of the full sample, the direction and significance of the results for the full sample did not change with either assumption (missing equal to zero or missing equal to one). The one exception was for laboratory tests, where the incremental effect of OSS was statistically significant, when missing data on the task was assumed to mean that it wasn’t performed (aRRR = 1.27, (95 % CI = 1.00–1.60)).

## Discussion

The trial demonstrated an incremental effect of OSS at improving patient history and physical examination, but not laboratory tests, diagnosis, treatment, or patients/caregiver education. These results for patient history were robust in sensitivity analyses with a balanced sample. Results for both patient history and physical examination were robust in an endline only comparison, and under alternative assumptions about missing values. There was also evidence that the IMID training program improved patient history and physical examination, but by the nature of the study design, we were unable to adjust for other changes that occurred at the facilities across time periods such as improved infrastructure, diagnostic capability etc. The effects of IMID were smaller and not statistically significant in the balanced sample. During OSS, a MLP was mentored one-on-one and what they previously learned in IMID was translated into their work environment. Regular reminders and applying principles to different patients resulted in better clinical practice.

Our results that IMID and OSS improved patient history and physical examination are consistent with the results from the IMCI Multi-Country Evaluation [14–16, 40]. We did not find strong evidence that IMID and OSS improved laboratory tests, diagnosis, treatment, and patient/caregiver education, whereas several evaluations of IMCI have shown effects on treatment and patient/caregiver education [15, 16, 40] [19]. There are three potential explanations for the absence of effects on these sets of tasks: 1) the intervention was not effective, 2) trainee practice on these tasks was higher at baseline than on patient history and physical examination, and had less room for improvement, and 3) the full effects were not measured because of the structure of the clinical assessment. Concerning the third explanation, the IDCAP clinical assessment was structured with an interruption after the patient history and physical examination when the clinical faculty completed or corrected them. From this point forward, the trainee had the results of a complete patient history and physical examination and may have been able to prescribe correct treatment and provide appropriate information to patients and caregivers. The intervention may have had larger and significant effects on these tasks if the clinical faculty did not intervene to complete the patient history and physical examination.

Horwood et al., in South Africa [41] conducted focus group discussions to establish whether skills gained after IMCI training were sufficient or whether additional follow up visits were required to maintain adequate skills. Findings from this South African study suggested that IMCI training course was an effective way to learn skills but follow-up was needed to improve implementation and retention of the skills. Facility based training as implemented in IMCI evaluations provided an opportunity to mentor health workers and is good value for money [40, 42].

Two strengths of the IDCAP clinical assessments were the relatively large number of observations per trainee, and the accurate patient information recorded by the clinical faculty member. Each trainee was observed an average of five times in each time period. The IDCAP clinical assessment was designed to observe the broad variety of children who present in a primary care facility. The patient history section was designed with follow-up questions as well as key questions, and the analysis accommodated differences in the number of appropriate questions across patients. The interruption after the patient history and physical examination ensured that emergency and complex cases benefited from the expertise of the clinical faculty, albeit having drawbacks as noted above.

### Limitations of this study

Firstly, two of the clinical faculty members at baseline did not conduct endline assessments and four new clinical faculty members conducted only endline assessments. To address this instability in measurement, we also analyzed the samples for whom the clinical faculty members were present both at baseline and endline and balanced across arms. The balanced sample however, no longer represented the full, randomized sample. Secondly, the missing information at baseline, especially for patient history and physical examination, could have an impact on the results. For the pre/post analysis, we may not be able to distinguish the effects of IMID in arm B from the more complete sample of patients in time 1. The sensitivity analyses however, showed that the results for patient history and physical examination were robust under two alternate assumptions about the missing values. The missing data analysis demonstrated the range of results with extreme values for the tasks that were used to construct each dependent variable. Multiple imputation of missing values would have been challenging, because the number of appropriate tasks varied across patients for four of the dependent variables. Thirdly, given that a 5 % level of significance was used despite multiple comparisons, it is possible that we erroneously concluded that the effects of the interventions were statistically significant. Fourthly, MLP were observed managing a convenience sample of patients rather than a random sample. It would be difficult to select patients at random in the absence of an appointment system. It’s unclear however, what how the selection process may have biased the results. Fifthly, trainees and observers did not know the allocation of facilities to arm during most of the baseline data collection, but they knew during the intervention and endline data collection. The clinical faculty did not observe their mentees at endline, but it’s possible that the observers were biased in favor of intervention arm. Finally, the clinical assessment data were limited to two time points at each facility, and don’t provide details of the monthly progress in clinical practice. To the extent that there were temporal trends in clinical practice, arm B controlled for them, so that the effect of OSS is measured accurately. The pre/post change in arm B however, could reflect these trends as well as the effects of IMID.

### Implications

Our results underscore the importance of continued on-site support or mentorship in addition to continuous medical education as a means to improve health worker practice. Task-shifting is a potentially cost-effective approach to addressing the human resource shortages in resource-limited settings. Programs that have embraced this approach should consider incorporating mentorship activities to improve the clinical practice of health workers and scaling up of health care services.

## Conclusions

On-site training and continuous quality improvement activities were associated with incremental improvements in patient history and physical examination among the MLP. This approach to capacity development may be more beneficial for developing countries especially as we move towards task shifting beyond HIV and AIDS care to management of common childhood infections. Companion articles will address the effects on clinical practice of HIV care, mortality among children less than five years of age and cost-effectiveness.

## Additional files

Additional file 1:
**Consort checklist for cluster randomised trials.** (DOCX 26 kb) Additional file 2:
**IDCAP outpatient clinical observation–child less than 5 years.** (PDF 151 kb) Additional file 3:
**Frequency of missing data on patient history.** (DOCX 17 kb) Additional file 4:
**Frequency of missing data in physical examination.** (DOCX 13 kb)

## Abbreviations

ART: Antiretroviral therapy
BIPAI: Baylor International Pediatric AIDS Initiative
CQI: Continuous quality improvement
HCI: Health Care Improvement Project
HIV: Human immunodeficiency virus
IDCAP: Integrated Infectious Disease Capacity-Building Evaluation
IDI: Infectious Diseases Institute
IMAI: Integrated Management of Adult Illness
IMCI: Integrated Management of Childhood Illness
IMID: Integrated management of infectious diseases
JUMP: Joint Uganda Malaria Program
MLP: Mid-level practitioner
OSS: On-site support
PMTCT: Prevention of mother-to-child transmission
RR: Relative risk
RRR: Ratio of relative risks
RTI: Respiratory tract infections
TB: Tuberculosis
