# Supplementary material for: Effect of integrated infectious disease training and on-site support on the management of childhood illnesses in Uganda: a cluster randomized trial
Source: BMC Pediatr. 2015 Aug 28;15:103. doi: 10.1186/s12887-015-0410-z (PMC4551363; doi:10.1186/s12887-015-0410-z)
Supplement: Additional file 1: — Consort checklist for cluster randomised trials. (DOCX 26 kb) [file 12887_2015_410_MOESM1_ESM.docx]

**Additional file 1. Consort checklist for cluster randomised trials**

| PAPER SECTION  and topic | Item | Descriptor | Reported on Page No. |
| --- | --- | --- | --- |
| TITLE & ABSTRACT | 1* | How participants were allocated to interventions (e.g., “random allocation”, “randomised”, or “randomly assigned”), *specifying that allocation was based on clusters* | 6 |
| INTRODUCTION  Background & Study Design | 2* | Scientific background and explanation of rationale, *including the rationale for using a cluster design.* | 4-6 |
| METHODS  Participants | 3* | Eligibility criteria for participants *and clusters* and the settings and locations where the data were collected. | 6-8, Miceli et al.,^[[1]](#endnote-1)^ Naikoba et al.^[[2]](#endnote-2)^ |
| Interventions | 4* | Precise details of the interventions intended for each group, *whether they pertain to the individual level, the cluster level or both,* and how and when they were actually administered. | 7-9,Miceli et al.,i Naikoba et al.ii |
| Objectives | 5* | Specific objectives and hypotheses, *and whether they pertain to individual, cluster level or both*. | 5-6, and 16 |
| Outcomes | 6* | Report clearly defined primary and secondary outcome measures, *whether they pertain to the individual level, the cluster level or both*, and, when applicable, any methods used to enhance the quality of measurements (e.g., multiple observations, training of assessors). | 10-12, Table 1 |
| Sample size | 7* | How *total* sample size was determined *(including method of calculation, number of clusters, cluster size, a coefficient of intracluster correlation (ICC or k), and an indication of its uncertainty*) and, when applicable, explanation of any interim analyses and stopping rules. | 13-14, Naikoba et al.ii |
| Randomisa-tion  Sequence generation | 8* | Method used to generate the random allocation sequence, including details of any restriction (e.g., blocking, stratification, *matching*). | 14-15 |
| Allocation concealment | 9* | Method used to implement the random allocation sequence, *specifying that allocation was based on clusters rather than individuals and* clarifying whether the sequence was concealed until interventions were assigned. | Naikoba et al.,ii Weaver et. al.^[[3]](#endnote-3)^ |
| Implemen-tation | 10 | Who generated the allocation sequence, who enrolled participants, and who assigned participants to their groups. | Naikoba et al.,ii Weaver et. al.iii |
| Blinding (Masking) | 11 | Whether or not participants, those administering the interventions, and those assessing the outcomes were blinded to group assignment. | 15 |
| Statistical methods | 12* | Statistical methods used to compare groups for primary outcome(s) *indicating how clustering was taken into account*; methods for additional analyses, such as subgroup analyses and adjusted analyses. | 15-18 |
| RESULTS  Participant flow | 13* | Flow of *clusters and* individual participants through each stage (a diagram is strongly recommended). Specifically, for each group report the numbers of *clusters and* participants randomly assigned, receiving intended treatment, completing the study protocol, and analyzed for the primary outcome. Describe protocol deviations from study as planned, together with reasons. | 19-20, Figure 1, Appendix Tables A & B  12-13 |
| Recruitment | 14 | Dates defining the periods of recruitment and follow-up. | 19 |
| Baseline data | 15* | Baseline information for each group *for the individual and cluster levels as applicable* | 20,  Table 2 |
| Numbers analyzed | 16* | Number of *clusters and* participants (denominator) in each group included in each analysis and whether the analysis was by “intention-to-treat”. State the results in absolute numbers when feasible (e.g., 10/20, not 50%). | 16-17  Figure 1, Tables 3-5 |
| Outcomes and Estimation | 17* | For each primary and secondary outcome, a summary of results for each group measures *for the individual or cluster level as applicable*, and the estimated effect size and its precision (e.g., 95% confidence interval) *and a coefficient of intracluster correlation (ICC or k) for each primary outcome.* | 21-23,  Table 3 |
| Ancillary analyses | 18 | Address multiplicity by reporting any other analyses performed, including subgroup analyses and adjusted analyses, indicating those pre-specified and those exploratory. | 22-13  Table 4 & 5 |
| Adverse events | 19 | All important adverse events or side effects in each intervention group. | Not applicable |
| DISCUSSION  Interpretation | 20 | Interpretation of the results, taking into account study hypotheses, sources of potential bias or imprecision and the dangers associated with multiplicity of analyses and outcomes. | 23-25 |
| Generalisa-bility | 21* | Generalisability (external validity) *to individuals and/or clusters (as relevant)* of the trial findings | 25-26 |
| Overall evidence | 22 | General interpretation of the results in the context of current evidence. | 27 |

1. Miceli A, Sebuyira LM, Crozier I, Cooke M, Omwangangye AP, Rayko-Farrar L, Ronald A, Tumwebaze M, Willis, Weaver MR. Advances in Clinical Education: A Model for Infectious Disease Training for Mid-Level Practitioners in Uganda. International Journal of Infectious Disease, 2012 (16): e708–e713 [↑](#endnote-ref-1)
2. Naikoba S, Colebunders R, Van Geertruyden JP, Willis KS, Kinoti SN, Mbonye MK, Mpanga-Sebuyira L, Ronald A, Scheld M, Weaver MR. Design of a cluster randomized trial assessing integrated infectious diseases training and onsite support for midlevel practitioners in Uganda. Journal of Clinical Care Pathways 2012; 16: 152-159. [↑](#endnote-ref-2)
3. Weaver MR, Crozier I, Eleku S, Makanga G, Sebuyira LM, Nyakake, Thompson ML, Willis K. Capacity-Building and Clinical Competence in Infectious Disease in Uganda: A Mixed-Design Study with Pre/Post and Cluster Randomized Trial Components. PLoS One 2012; 7(12):e51319. [↑](#endnote-ref-3)
