# Supplementary material for: Effect of integrated infectious disease training and on-site support on the management of childhood illnesses in Uganda: a cluster randomized trial
Source: BMC Pediatr. 2015 Aug 28;15:103. doi: 10.1186/s12887-015-0410-z (PMC4551363; doi:10.1186/s12887-015-0410-z)
Supplement: Additional file 2: — IDCAP outpatient clinical observation–child less than 5 years. (PDF 151 kb) [file 12887_2015_410_MOESM2_ESM.pdf]

A. **Observation #:___________________**

IDCAP Outpatient Clinical Observation – Child less than 5 years

B. Site #:	C. Trainee #:	D. Date of visit (d/m/y):
E. Observer #:		F. Quality control #:
G. **Triage status:** ☐ H. Emergency (☐A ☐B ☐C ☐D ☐O) ☐ I. Priority (Specify_______________) ☐ Not Emergency		
**If emergency, support trainee to manage patient. Emergency treatment is higher priority than clinical assessment.**		

J. Language of patient during visit: ________ K. Translation? ☐Yes ☐No

L. Gender of patient: ☐F ☐M M. Age of patient: _______ months N. Date of birth: (d/m/y):___________________

**I. Vital signs:** 1. Reported by other health professional or volunteer before consultations? ☐Yes ☐No	
2. Temperature **_____C** 3. If no thermometer, febrile to touch ☐Y ☐N<br>4. Current weight **____ kg** 5. Height**____cm** 6. Weight last visit **____kg** 7. Last visit(d/m/y):___/___/___	☐<br>☐

II. History

1. **Type of visit:** ☐New attendance ☐Re-attendance

What are the child's complaints? 2. ☐cough/difficulty breathing 3. ☐diarrhea 4. ☐ear pain 5. ☐fever 6. ☐HIV exposed 7. ☐vomiting 8. ☐other (Specify)_________________________________

9. **Does the patient have danger signs?** ☐Y ☐N ☐NR If yes, specify 10. ☐Change in consciousness/lethargic 11. ☐Convulsions 12. ☐Not able to drink/breastfeed 13. ☐Vomits everything 14. ☐Other___________________

	Patient	Code		Patient	Code
15. **Fever?**	☐Y ☐N ☐NR	☐	29. **Ear pain?**	☐Y ☐N ☐NR	☐
16. Duration	___days ☐NR ☐NA	☐	30. Duration	___days ☐NR ☐NA	☐
17. Measles within the last 3 months?	☐Y ☐N ☐NR ☐NA	☐	31. **Ear discharge?**	☐Y ☐N ☐NR	☐
18. **Coughing?**	☐Y ☐N ☐NR	☐	32. Duration	___days ☐NR ☐NA	☐
19. Duration	__days ☐NR ☐NA	☐	33. **HIV status mother?**	☐Y ☐N ☐NR	☐
20. **If yes to Fever or Cough**			34. If Y, did mother PMTCT?	☐Y ☐N ☐NR ☐NA	☐
21. Night sweats?	☐Y ☐N ☐NR ☐NA	☐	35. Optional: HIV Status child?	☐Y ☐N ☐NR ☐NA	☐
22. Weight loss?<br>23. Specify % _____	☐Y ☐N ☐NR ☐NA	☐	36. **Diarrhea?**	☐Y ☐N ☐NR	☐
24. Recent contact with someone who has TB?	☐Y ☐N ☐NR ☐NA	☐	37. Duration	___days ☐NR ☐NA	☐
25. **Medication before today's visit?**<br>26. If yes, specify	☐Y ☐N ☐NR<br>____________________	☐	38. Blood?	☐Y ☐N ☐NR ☐NA	☐
27. **Immunization up to date?**	☐Y ☐N ☐NR	☐	39. **Vitamin A in last 6 months?**	☐Y ☐N ☐NR	☐
28. Confirm with card?	☐Y ☐N ☐NR ☐NA	☐	40. Confirm with card?	☐Y ☐N ☐NR ☐NA	☐
41. **Other current symptoms or concerns?** 42. If yes, specify ____				☐Y ☐N ☐NR	☐

43. Did equipment or resource gaps affect trainee's history taking for this patient? ☐Yes ☐No

44. If yes, please explain____

III. Physical examination			
1. Danger Sign		☐	A.☐none B.☐drinks eagerly/thirsty C.☐lethargic D.☐not able to drink or breastfeed
2. Growth		☐	A.☐normal B.☐low weight for age against weight chart C.☐low weight for age not improving D.☐missed or lost developmental milestone
3. General		☐	A.☐normal B.☐palor C.☐jaundice D.☐oedema E.☐lymphadenopathy F.☐eyes, red G.☐eyes, sunken H.☐abnormal pinch skin I.☐runny nose J.☐restless, irritable K.☐wasting L.☐temperature______________**C** M.☐other_____________________
4. Mouth		☐	A.☐normal B.☐sores C.☐oral thrush D.☐caries E.☐white spots on throat F.☐other ___________
5. Ear		☐	A.☐normal B.☐pus/drainage C.☐swelling behind ear D.☐ inflamation of ear drum E.☐other _______________
6. Skin		☐	A.☐normal B.☐generalized rash C. ☐other_____________________________
7. Lungs		1.☐ 2.☐ 3.☐	A.☐normal B.☐breathing difficulty C. ☐chest in-drawing D.☐stridor<br>If cough, E.☐RR–trainee_________bpm F.☐RR–observer______________bpm<br>Listen to lung: G.☐clear H.☐abnormal sound on percussion I.☐tenderness J.☐crepitations K.☐rhonchi L.☐ wheezing M.☐decreased breath sounds N.☐other___________
8. Abdomen		☐	A.☐normal B.☐distended C.☐tenderness D.☐abnormal sound on percussion E.☐hepatomegaly F.☐splenomegaly G.☐abnormal mass H.☐other ___________
9. CNS		☐	A.☐normal B.☐impaired level of consciousness C.☐abnormal muscle tone D.☐abnormal reflexes E.☐stiff neck F.☐central neural deficit G.☐peripheral neural deficit H.☐other___________________________________
10. Other- Specify: _______		☐	A.☐normal B.☐Specify findings___________________________
10. Did equipment or resource gaps affect trainee's physical examination for this patient? ☐Yes ☐No<br>11. If yes, please explain____			

VII1. Did trainee conduct a focused and thorough history that is relevant to evolution of current symptom/complaint? ☐Yes ☐No VII2. If no, summarize reason.

☐Omission (Specify if not obvious on checklist)

☐Misinterpretation (Must specify)

☐Unnecessary (Must specify)

VII3. Did trainee conduct a complete physical exam? ☐Yes ☐No VII4. If no, summarize reason.

☐Omission (Specify if not obvious on checklist)

☐Misinterpretation (Must specify)

☐Unnecessary (Must specify)

Site/trainee/Observation#_____/_____/_____

IV. Investigations

What laboratory investigations would you order today? 1. ☐ None					
Code		Results	Code		Results
	2. ☐ Ab (HIV antibody)			3. ☐ Ag/PCR (child< 18mo)	
	4. ☐ CBC (hemogram)			5. ☐ HB (haemoglobin)	
	6. ☐ Malaria BS			7. ☐ Malaria RDT	
	8. ☐ RPR			9. ☐ Stool	
	10. ☐ TB sputum			11. ☐ Urinalysis	
	12. ☐ Other1- Specify _______________			13. ☐ Other2- Specify _________________	

14. Would you order other investigations or procedures today? ☐ Yes ☐ No If yes, specify below.					
Code		Results	Code		Results
	15. ☐ Chest x-ray			16. ☐ Ultrasound scan Specify______________	
	17. ☐ Other x-ray - Specify ________			18. ☐ Other3-Specify ___________________	
19. Did equipment or resource gaps affect investigations for this patient? ☐ Yes ☐ No<br>20. If yes, please explain____					

VII5. Did trainee recommend appropriate investigations? ☐ Yes ☐ No VII6. If no, summarize reason.

☐ Omission (Specify if not obvious on checklist)

☐ Misinterpretation (Must specify)

☐ Unnecessary (Must specify)

V. What are your diagnoses?					
Code		Code		Code	
	1. ☐ One or more danger signs<br>2. ☐ Anaemia – mild<br>3. ☐ Anaemia - moderate<br>4. ☐ Anaemia - severe<br>5. ☐ Cough (no pneumonia)<br>6. ☐ Diarrhea – acute<br>7. ☐ Diarrhea – persistent<br>8. ☐ Dysentery		9. ☐ Ear - Otitis media (pus)<br>10. ☐ Ear - Other infection<br>11. ☐ HIV infection<br>12. ☐ HIV suspect<br>13. ☐ Malaria (uncomplicated)<br>14. ☐ Malaria (complicated)<br>15. ☐ Malnutrition (low weight for age)<br>16. ☐ Severe malnutrition		17. ☐ Measles<br>18. ☐ Meningitis<br>19. ☐ Pneumonia<br>20. ☐ TB Disease – pulmonary<br>21. ☐ TB Disease-extra pulmonary<br>22. ☐ TB suspect<br>23. ☐ Other – Specify<br>________________________<br>________________________

VII7. Did trainee accurately made appropriate diagnosis? ☐ Yes ☐ No VII8. If no, summarize reason.

☐ Omission (Specify if not obvious on checklist)

☐ Misinterpretation (Must specify)

☐ Unnecessary (Must specify)

VI. What treatment would you recommend?

1. **Malaria Tx?:** ☐Yes ☐No If yes, specify<br>3. Treatment 1? ____________________<br>5. Route: ☐oral ☐parenteral ☐Specify __________<br>7. Treatment 2____________________<br>9. Route: ☐oral ☐parenteral ☐Specify __________	2. **Observer agree w/malaria Tx?** ☐Yes ☐No, specify<br>4. Treatment 1? ____________________<br>6. Route: ☐oral ☐parenteral ☐Specify __________<br>8. Treatment 2____________________<br>10. Route: ☐oral ☐parenteral ☐Specify __________
11. **Antibiotic Tx?** ☐Yes ☐No If yes, specify<br>13. Treatment 1? ____________________<br>15. Route: ☐oral ☐parenteral ☐Specify ________<br>17. Treatment 2____________________<br>19. Route: ☐oral ☐parenteral ☐Specify ________	12. **Observer agree w/antibiotic Tx?** ☐Yes ☐No, specify<br>14. Treatment 1? ____________________<br>16. Route: ☐oral ☐parenteral ☐Specify __________<br>18. Treatment 2____________________<br>20. Route: ☐oral ☐parenteral ☐Specify __________
21. **Other Tx ?** ☐Yes ☐No If yes, specify<br>23. Treatment 1? ____________________<br>25. Route: ☐oral ☐parenteral ☐Specify ________<br>27. Treatment 2____________________<br>29. Route: ☐oral ☐parenteral ☐Specify ________	22. **Observer agree with other Tx?** ☐Yes ☐No, specify<br>24. Treatment 1? ____________________<br>26. Route: ☐oral ☐parenteral ☐Specify __________<br>28. Treatment 2____________________<br>30. Route: ☐oral ☐parenteral ☐Specify __________
31. **Internal referral or consult?:** ☐Yes ☐No<br>33. Who? ____________________<br>35. Reason? ____________________<br>37. **External referral:** ☐Yes ☐No<br>39. Where? ____________________<br>41. Reason? ____________________<br>43. **Admitted this site:** ☐Yes ☐No<br>45. **Date of next visit: ____/_____/________**	32. **Observer agree w/ internal referral or consult:**<br>☐Yes ☐No , Specify. 34. Who?____________________<br>36. Reason?____________________<br>38. **Observer agree w/ external referral or consult:**<br>☐Yes ☐No, Specify. 40. Who?____________________<br>42. Reason?____________________<br>44. **Observer agree w/admission?** ☐Yes ☐No<br>46. **Observer agree w/ Date of next visit?** ☐Yes ☐No

**Prevention provided?**		**Parent/adult given information on?**	
47. CTX prophylaxis	☐Yes ☐No ☐NA	51. Diagnosis	☐Yes ☐No ☐NA
48. Recommend Mosquito Net	☐Yes ☐No	52. Explain treatment	☐Yes ☐No ☐NA
49. Recommend Vitamin A	☐Yes ☐No ☐NA	53. Provide instructions on how to complete treatment	☐Yes ☐No ☐NA
50. Update immunizations	☐Yes ☐No ☐NA		

54. Did equipment or resource gaps affect trainee's treatment plan for this patient? ☐Yes ☐No
55. If yes, please explain____________________

**Key: Antimalarials prescribed**		
1. Amodiaquine (AQ)	4. Dihydroartemisinin + Piperaquine (Duocotexin/Artekin) (DP)	7. Sulfadoxine Pyrimethamine (SP)
2. Artesunate + Amodiaquine (AQ+AS)	5. Chloroquine +Fansidar	8. Artemether + Lumefantrine (Coartem)
3. Quinine (QN)	6. Chloroquine (CQ	9. Artesunate (AS)
		10. Artemether
**Key: Antibiotics prescribed**		
1. Amoxicillin	5. Chloramphenicol	10. Gentamicin
2. Ampicillin	6. Ciprofloxacin	11. Metronidazole
3. Benzyl Penicillin	7. Cotrimoxazole	12. PPF (Fortified Procaine Penicillin)
4. Ceftriaxone	8. Doxycycline	13. Tetracycline
	9. Erthromycin	

VII9. Did trainee recommend appropriate drug treatment? ☐Yes ☐No VII10. If no, summarize reason.

☐Omission (Specify if not obvious on checklist)

☐Misinterpretation (Must specify)

☐Unnecessary (Must specify)
