# Supplementary material for: Effect of integrated infectious disease training and on-site support on the management of childhood illnesses in Uganda: a cluster randomized trial
Source: BMC Pediatr. 2015 Aug 28;15:103. doi: 10.1186/s12887-015-0410-z (PMC4551363; doi:10.1186/s12887-015-0410-z)
Supplement: Additional file 3: — Frequency of missing data on patient history. (DOCX 17 kb) [file 12887_2015_410_MOESM3_ESM.docx]

| Additional File 3: Frequency of missing data on patient history | | | | | | |
| --- | --- | --- | --- | --- | --- | --- |
|  | Baseline | | | Endline | | |
|  | Phase A | Phase B | Total | Phase A | Phase B | Total |
|  | N=161  n (%) | N=176  n (%) | N=337  n (%) | N=177 n (%) | N=173  n (%) | N=350  n (%) |
| Checked for danger sign | 20 (12) | 15 (9) | 35 (10) | 6 (3) | 2 (1) | 8 (2) |
| Checked for fever | 1 (1) | 0 (0) | 1 (0.3) | 1 (1) | 0 (0) | 1 (0.3) |
| If fever |  |  |  |  |  |  |
| Duration | 1 (1) | 4 (3) | 5 (2) | 0 (0) | 0 (0) | 0 (0) |
| Prior antimalarials | 6 (5) | 6 (4) | 12 (4) | 0 (0) | 1 (1) | 1 (0.3) |
| Measles in last 3 mo | 24 (18) | 29 (19) | 53 (19) | 8 (5) | 5 (4) | 13 (4) |
| Ear pain | 3 (2) | 4 (3) | 7 (2) | 0 (0) | 0 (0) | 0 (0) |
| Checked for ear discharge | 4 (2) | 3 (2) | 7 (2) | 1 (1) | 2 (1) | 3 (1) |
| If Ear discharge |  |  |  |  |  |  |
| Duration | 1 (11) | 0 (0) | 1 (8) | 0 (0) | 0 (0) | 0 (0) |
| Checked for HIV status | 11 (7) | 3 (2) | 14 (4) | 0 (0) | 0 (0) | 0 (0) |
| Checked for cough | 0 (0) | 1 (1) | 1 (0.3) | 1 (1) | 0 (0) | 1 (0.3) |
| If cough |  |  |  |  |  |  |
| Duration | 6 (7) | 1 (1) | 7 (3) | 2 (2) | 2 (2) | 4 (2) |
| If cough>14 days |  |  |  |  |  |  |
| Night sweats | 1 (25) | 0 (0) | 1 (10) | 0 (0) | 0 (0) | 0 (0) |
| Weight loss | 1 (25) | 0 (0) | 1 (10) | 1 (13) | 0 (0) | 1 (8) |
| Recent contact | 1 (25) | 0 (0) | 1 (10) | 1 (13) | 0 (0) | 1 (8) |
| Checked for diarrhea | 1 (1) | 2 (1) | 3 (1) | 0 (0) | 1 (2) | 1 (1) |
| If diarrhea |  |  |  |  |  |  |
| Duration asked | 2 (4) | 1 (2) | 3 (3) | 0 (0) | 2 (4) | 2 (2) |
| Blood in stool asked | 9 (19) | 7 (13) | 16 (16) | 0 (0) | 0 (0) | 1 (1) |
| Checked for immunization (age <5yr) | 7 (5) | 8 (5) | 15 (5) | 2 (1) | 3 (2) | 5 (1.5) |
| Checked for other problems | 13 (8) | 14 (8) | 27 (8) | 1 (1) | 1 (1) | 2 (1) |

Danger signs and measles status were not included in the full and balanced sample comparisons
