# Supplementary material for: Effect of integrated infectious disease training and on-site support on the management of childhood illnesses in Uganda: a cluster randomized trial
Source: BMC Pediatr. 2015 Aug 28;15:103. doi: 10.1186/s12887-015-0410-z (PMC4551363; doi:10.1186/s12887-015-0410-z)
Supplement: Additional file 4: — Frequency of missing data in physical examination. (DOCX 13 kb) [file 12887_2015_410_MOESM4_ESM.docx]

| Additional File 4: Frequency of missing data in physical examination | | | | | | |
| --- | --- | --- | --- | --- | --- | --- |
|  | Baseline | | | Endline | | |
|  | Phase A | Phase B | Total | Phase A | Phase B | Total |
|  | N=161 n (%) | N=176  n (%) | N=338  n (%) | N=177 n (%) | N=173  n (%) | N=150 n (%) |
| 1. Danger Sign | 3 (2) | 2 (1) | 5 (1.5) | 0 (0) | 0 (0) | 0 (0) |
| 2. General | 2 (1) | 2 (1) | 4 (1) | 0 (0) | 0 (0) | 0 (0) |
| 3. Skin | 6 (4) | 7 (4) | 13 (4) | 0 (0) | 0 (0) | 0 (0) |
| 4. Lungs | 7 (4) | 2 (1) | 9 (3) | 0 (0) | 0 (0) | 0 (0) |
| 5. Abdomen | 4 (2) | 1 (1) | 5 (1.5) | 0 (0) | 0 (0) | 0 (0) |
| 6. Growth | 48 (10) | 48 (18) | 96 (14) | 0 (0) | 0 (0) | 0 (0) |
| 7. Mouth | - | - | - | 0 (0) | 0 (0) | 0 (0) |
| 8. Ear | 8 (5) | 5 (3) | 13 (4) | 0 (0) | 0 (0) | 0 (0) |
| 9. Central nervous system | 12 (7) | 11 (6) | 23 (7) | 0 (0) | 0 (0) | 0 (0) |
| 10. Other | 96 (59) | 103 (59) | 199 (59) | 120 (68) | 128 (74) | 248 (71) |
| Systems 1-5, 8 and 9 were used in full and balanced sample comparisons. “Other” system was not included when “other” data were missing; i.e. it was not counted in the numerator or the denominator.  All systems (1-9) were used only in endline sample comparisons. “Other system was not included when “other” data were missing, as described above. | | | | | | |
